# Cushing syndrome as a failed cardiac screen in a patient with McCune–Albright syndrome: a case report

**DOI:** 10.1186/s13256-022-03533-1

**Published:** 2022-09-15

**Authors:** Christy Foster, Hiba Al Zubeidi, Alicia Diaz-Thomas

**Affiliations:** 1 Division of Endocrinology, Department of Pediatrics, University of Alabama at Birmingham, 1601 4th Avenue South, Birmingham, AL 35233 USA; 2 Division of Endocrinology, Department of Pediatrics, University of Tennessee Health Science Center, Memphis, TN USA

**Keywords:** Cushing syndrome, Cardiac hypertrophy, McCune–Albright syndrome

## Abstract

**Background:**

McCune–Albright syndrome is a complex disorder encompassing multiple endocrinopathies. These manifestations are secondary to a mutation in the stimulatory G-protein alpha subunit. Cushing syndrome is due to autonomous secretory function of the adrenal gland and is present in 7.1% of patients with McCune–Albright syndrome. Cardiac newborn screenings assist in the identification of critical congenital heart disease. These screenings have become part of routine postnatal care nationwide.

**Case report:**

A 6-week-old Caucasian male presented to a cardiologist at the University of Tennessee Health Science Center with left ventricular hypertrophy and poor feeding after a failed cardiac newborn screen. He had been previously seen at 2 weeks by a cardiologist on follow-up for abnormal critical congenital heart disease screening. Electrocardiogram and echocardiographic studies identified hypertrophic cardiomyopathy. Other examination findings revealed multiple characteristic café-au-lait lesions along with hypotonia and rounded facies. Given his cardiac disease, he was admitted to the hospital, where an evaluation was done for Cushing syndrome, showing elevated cortisol by immunoassay of 38 μg/dL (1.7–14.0 μg/dL, Vitros 5600) after a dexamethasone suppression test and urinary cortisol elevated to 35 μg/dL/24 hours (reference range 3–9 μg/dL/24 hours) (Esoterix; Calabasas, CA). He was started on metyrapone therapy to block synthesis of cortisol. His cortisol improved and was suppressed less than 2 μg/dL. His hypertension and clinical features of Cushing syndrome improved.

**Conclusions:**

This case demonstrates a unique presentation of Cushing syndrome in a young infant. This is the first case to our knowledge showing significant left ventricular hypertrophy resulting from Cushing syndrome identified following a failure on a critical congenital heart disease screen. It highlights the importance of considering of McCune–Albright syndrome in patients with Cushing syndrome, especially if other clinical features are present. Medical therapy can be used to treat Cushing syndrome and can result in improvement in the cardiovascular pathology.

## Background

Neonatal screening for congenital heart disease has been implemented to identify critical congenital heart disease such as hypoplastic left heart, transposition of the great arteries, and tetralogy of Fallot [1]. Hypertrophic cardiomyopathy can be identified on a newborn heart screen if it is severe enough to cause outflow obstruction and therefore is considered in the differential of a failed cardiac screen [2].

McCune–Albright syndrome (MAS) is a condition characterized by café-au-lait lesions and hyperfunctional endocrinopathies [3, 4]. The manifestations are due to mutations in the stimulatory G-protein alpha subunit caused by a postzygotic mutation in *GNAS1*, leading to activation of the alpha-G protein subunit [5]. MAS is often mosaic, with increased mosaicism resulting in a greater disease burden. Cushing syndrome (CS) in the setting of MAS, most often in younger patients, reflects an activation of *GNAS* gene expression involving the adrenal cortex. The adrenocorticotropic hormone (ACTH)-independent activation of adrenocortical cells has previously been reported in infancy [6–8]. A National Institutes of Health (NIH) study showed that the prevalence of CS was 7.1% among patients with MAS who are followed at the NIH, which often includes the most significantly affected.

Treatment for CS can range from medical therapy and close monitoring to bilateral adrenalectomy in the case of severe disease. In one series, 10 out of 23 patients had spontaneous resolution of their CS [9]. Given the low number of reported patients, it can be difficult to recognize which patients can be safely monitored or treated medically versus those patients who need adrenalectomy. According to the NIH study, comorbid heart and liver disease were poor prognostic indicators. Left ventricular hypertrophy (LVH) and cardiomegaly have been noted in patients with CS [10, 11]. For patients with MAS, the pathology of the cardiomyopathy is secondary to the prolonged exposure to excess cortisol in CS.

We report on an infant with MAS who presented with cardiac pathology secondary to Cushing syndrome and describe treatment with metyrapone and resulting improvement in hypertrophic cardiomyopathy.

## Case presentation

### Initial presentation

This Caucasian patient was born at 36 weeks gestation via vaginal delivery to a gravida 2, para 2 mother with no significant past medical history. His initial birth weight was 2381 g (25–50th percentile). He failed his newborn pulse oximetry cardiac screen and was first seen by cardiology at 2 weeks of age. He was followed in the cardiology clinic again at 1 month of age. At that time, an electrocardiogram (EKG) showed a sinus tachycardia of 200 beats per minute and hypertension. An echocardiogram was significant for left ventricular hypertrophy with septal wall thickness in diastole of 7 mm and a posterior wall measurement of 5 mm consistent with left ventricular noncompaction, hypertrophic cardiomyopathy subtype. He was also noted to have different skin tones and pigmentation from trunk to lower extremities. He was started on a beta-blocker with plans to follow up in clinic in 2 months and was referred to dermatology for evaluation of the skin lesions.

At 6 weeks of age, he presented to the cardiomyopathy clinic owing to left ventricular hypertrophy and was also having difficulty feeding. He was then admitted to the hospital for failure to thrive. At admission, his weight was 3100 g (< 2nd percentile), length was 48 cm (3rd–10th percentile), and head circumference was 34 cm (< 2nd percentile). His physical examination findings were significant for hypertension (101/68 mmHg), tachycardia, facial fullness, hyperpigmented lesions on his skin, marked hypotonia, and hepatomegaly. He had irregular hyperpigmentation characterized as a swirling pattern over the back measuring approximately 5 × 2 cm in area under the right axilla and extending to the right portion of the anterior trunk, stopping at the midline.

Laboratory findings demonstrated transaminitis: aspartate transaminase (AST) 99 U/L (normal 22–63 U/L); alanine transaminase (ALT) 250 U/L (normal 13–39 U/L); total bilirubin 2.3 mg/dL (normal 0.2–1.0 mg/dL).

**Fig. 1:**
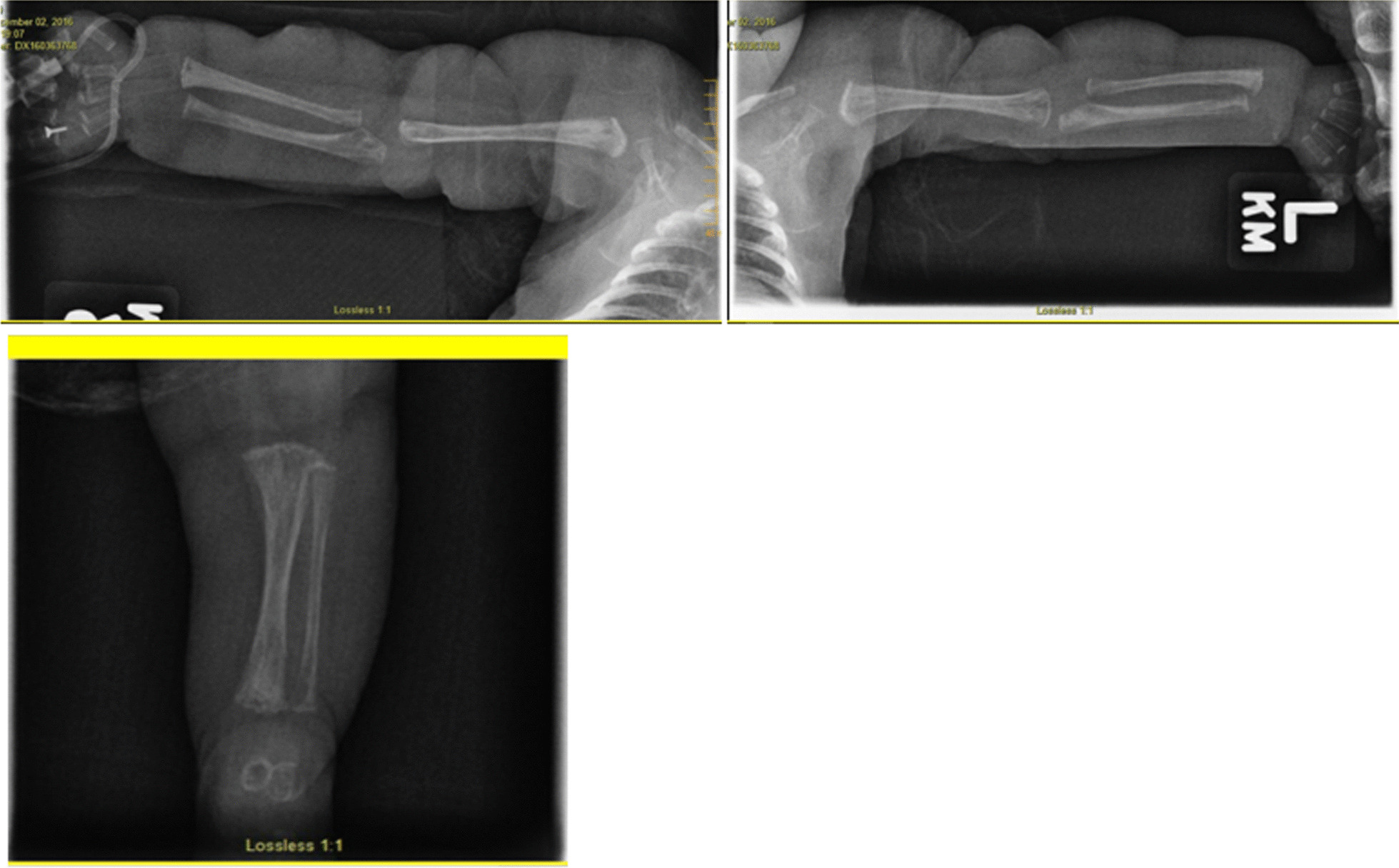
Skeletal survey demonstrating polyostotic fibrous dysplasia; top left, right upper extremity; top right, left upper extremity; bottom left, left femur

A chest radiograph raised concern for cardiomegaly, and an osseous x-ray showed multiple lesions of polyostotic fibrous dysplasia (Fig. 1) Genetics was consulted during the hospital admission. The diagnosis of MAS was made on the basis of the findings of adrenal fullness on a renal ultrasound in conjunction with the hyperpigmented lesion, polyostotic fibrous dysplasia, cardiomyopathy, and elevated liver function tests  (LFT). Repeat echocardiograms showed persistent concentric left ventricular hypertrophy, with initial thickness of the left ventricular wall of 4.7 mm (*Z*-score 2.6) reaching a maximum of 7.1 mm (*Z*-score 1.7) (Table 1). Systemic hypertension was confirmed with systolic pressures up to 130 mmHg and diastolic pressures up to 77 mmHg.

**Table 1:** Measurement of left posterior wall thickness (LV PW) on each echocardiogram

	LV PW (mm)	LV PW *Z*-score	Ejection fraction (%)	LV septum (mm)	
Initial echocardiogram (30 November 2016)	4.7–7.1	2.60	69.6	5.6–6.6	2.05
Most recent echocardiogram (5 May 2020)	5.1–7.9	0.85	66.8	4.6–7.6	−0.32

*LV* left ventricle, *PW* posterior wall, *mm* millimeter

A renal ultrasound was negative for renal vein anomalies, and metanephrines were also found to be normal. A random cortisol done via immunoassay was found to be 36.4 μg/dL (1.7–14.0 μg/dL, Vitros 5600) Total protein, albumin, prealbumin, and creatinine kinase were normal. An abdominal ultrasound showed increased liver echogenicity, bilaterally enlarged adrenal glands, and bilateral nephrocalcinosis.

Given the likelihood of MAS, evaluation for CS was performed. Biochemical evaluation showed elevated serum cortisol without diurnal variation (8 a.m. cortisol at 38.2 μg/dL and 7 p.m. cortisol at 36.4 μg/dL; normal 2.8–23.0 μg/dL). Urinary free cortisol was elevated to 35 μg/dL/24 hours (reference range 3–9 μg/dL/24 hours) (Esoterix; Calabasas, CA). A low-dose dexamethasone suppression test revealed a cortisol of 38.2 μg/dL and an ACTH level of < 5 pg/mL; this was followed by a high-dose dexamethasone suppression test that showed a serum cortisol of 60.1 μg/dL. These results showed that failure to suppress cortisol was consistent with ACTH-independent CS.

A skin biopsy of a café-au-lait lesion on his back was obtained for GNAS gene analysis at All Children’s Hospital in St. Petersburg, Florida and was negative. This result does not negate the clinical diagnosis of MAS given the variable expression of the mutation (somaticism) that can occur.

### Treatment and management

Our patient underwent medical treatment of CS with metyrapone. We obtained initial suppression of his cortisol to < 10 μg/dL after titration to a dose of 18.5 mg/kg divided twice daily.

Given his failure to thrive and oral aversion, a G-tube was placed for nutritional support. He continued to have motor developmental delay; however, with control of his CS, his hypotonia improved and he was able to participate in physical therapy, sitting up, rolling over, and eventually taking steps with a walker. He is currently independently walking and starting to run.

Hypertensive control required the combination of two antihypertensives (clonidine and metoprolol). His echocardiogram has shown improvement in left ventricular hypertrophy. He has had normal left ventricular wall thickness at 6 mm and ejection fraction of 63%. His blood pressure was 83/49 mmHg.

### Patient course

To date, at 27 months of age, he has not been found to have any signs of growth hormone excess or thyroid excess but has signs of peripheral precocious puberty that can be seen in MAS. His cardiomyopathy has improved with treatment of his CS. Close monitoring of his growth has revealed a normal growth velocity. Periodic thyroid function tests have been normal.

## Conclusions

We describe a case of MAS presenting with CS at 6 weeks of age that was identified owing to failure of newborn pulse oximetry and abnormal cardiac findings. Other previously reported neonatal patients with MAS have often presented with polyendocrine disease, bone disease, nephrocalcinosis, and even intrauterine growth restriction (IUGR) at birth, likely secondary to earlier mutations in embryogenesis [6, 12, 13].

This patient’s presentation entailed a failed cardiac newborn screen. This screen shows a sensitivity of 95% for congenital heart disease. As discussed previously, hypertrophic cardiomyopathy can be identified on a newborn heart screen if it is severe enough to cause outflow obstruction and therefore is on the list of potential cardiac anomalies to consider in the differential diagnosis of a failed cardiac screen [2]. CS should be considered on the basis of hypertrophic cardiomyopathy and intractable hypertension diagnosis, especially in patients who have other concerning features.

Clinical manifestations for CS can vary widely, from mild symptoms with spontaneous resolution to intractable hypercortisolism leading to a severe phenotype. Classic treatment has involved adrenalectomy to prevent symptoms of hypertension and hypertransaminitis. According to Endocrine Society guidelines, bilateral adrenalectomy is recommended as first-line treatment [14]. Several cases have demonstrated that CS can resolve spontaneously in patients with MAS [7, 9]. After consultation with the medical team and the family, we elected to purse medical theory with close follow-up to avoid risks involved with surgery.

In our case, after treatment of his CS, our patient showed improvement in hypertension and interventricular septum thickness. Prior reports have shown improvement in cardiac disease in those treated for their CS [15–17]. This is the first case, to our knowledge, to show significant LVH resulting from CS leading to a failed newborn screen. Our case also demonstrates that medical therapy for Cushing syndrome is a reasonable option for an infant with MAS in select cases where surgery is avoided to improve their cardiovascular disease.

## Data Availability

Not applicable.

## Abbreviations

MAS: McCune–Albright syndrome
CS: Cushing syndrome
LVH: Left ventricular hypertrophy
